# Human cerebrospinal fluid contains CD4+ memory T cells expressing gut- or skin-specific trafficking determinants: relevance for immunotherapy

**DOI:** 10.1186/1471-2172-7-14

**Published:** 2006-07-07

**Authors:** Pia Kivisäkk, Barbara Tucky, Tao Wei, James J Campbell, Richard M Ransohoff

**Affiliations:** 1 Neuroinflammation Research Center, Department of Neurosciences, Lerner Research Institute, The Cleveland Clinic, 9500 Euclid Ave, Cleveland, OH 44195, USA; 2 Joint Program in Transfusion Medicine, Children's Hospital and Department of Pathology, Harvard Medical School, 300 Longwood Ave, Boston, MA 02115, USA; 3 Center for Neurologic Diseases, Brigham and Women's Hospital and Department of Neurology, Harvard Medical School, 77 Ave Louis Pasteur, Boston, MA 02115, USA

## Abstract

**Background:**

Circulating memory T cells can be divided into tissue-specific subsets, which traffic through distinct tissue compartments during physiologic immune surveillance, based on their expression of adhesion molecules and chemokine receptors. We reasoned that a bias (either enrichment or depletion) of CSF T cell expression of known organ-specific trafficking determinants might suggest that homing of T cells to the subarachnoid space could be governed by a CNS-specific adhesion molecule or chemokine receptor.

**Results:**

The expression of cutaneous leukocyte antigen (CLA) and CC-chemokine receptor 4 (CCR4; associated with skin-homing) as well as the expression of integrin α4β7 and CCR9 (associated with gut-homing) was analyzed on CD4+ memory T cells in CSF from individuals with non-inflammatory neurological diseases using flow cytometry. CSF contained similar proportions of CD4+ memory T cells expressing CLA, CCR4, integrin α4β7 and CCR9 as paired blood samples.

**Conclusion:**

The results extend our previous findings that antigen-experienced CD4+ memory T cells traffic through the CSF in proportion to their abundance in the peripheral circulation. Furthermore, the ready access of skin- and gut-homing CD4+ memory T cells to the CNS compartment *via *CSF has implications for the mechanisms of action of immunotherapeutic strategies, such as oral tolerance or therapeutic immunization, where immunogens are administered using an oral or subcutaneous route.

## Background

The differentiation of naïve T cells into an activated memory phenotype is characterized by an extensive change in the expression of trafficking determinants, resulting in the acquisition of homing receptors that enable the cells to migrate from the circulation into peripheral tissues. This change in T cell homing potential is affected by the microenvironment where initial antigen recognition occurred, as memory T cells preferentially return to regions of the body similar to those where the initial antigen was encountered [1]. For instance, studies in mice have demonstrated that CD4+ T cells activated in cutaneous lymph nodes upregulate trafficking determinants specific for the skin, such as P-selectin ligand, while T cells responding to antigen in intestinal lymph nodes express high levels the gut-associated adhesion molecule integrin α4β7 and acquire responsiveness to the intestinal CC-chemokine ligand CCL25 [2]. The specific profile of adhesion molecules and chemoattractant receptors expressed by individual T cells allows the cells to interact with the vascular endothelium at anatomical sites where the cognate ligands are selectively expressed, targeting the cells to specific tissues [3,4]. The relevance of such interactions has been amply demonstrated in humans, where postcapillary venules in the inflamed skin selectively express E-selectin and CCL17, while lamina propria of the small intestines displays the mucosal addressin MAdCAM-1 and CCL25 [5-7]. Interestingly, T cells isolated from the small intestines and the skin are almost completely dichotomous in their expression of trafficking determinants; T cells from the skin express cutaneous leukocyte antigen (CLA) and CCR4, but lack integrin α4β7, while T cells from the small intestines are positive for α4β7, but not CLA or CCR4 [6,8-10]. Consequently, circulating T cells can be divided into tissue specific subsets, each of which have the ability to traffic through certain tissue compartments, but which are excluded from others.

While trafficking of T cells to the skin and gut is well characterized, less is known about the mechanisms governing homing of T cells across the choroid plexus into the CSF during immune surveillance of the healthy brain [11]. Although it has been hypothesized that such CSF-specific trafficking determinants exist, their molecular specificity has been evasive. Some progress has been made in defining trafficking determinants for inflamed CNS microvessels: CD8+ T cells activated in cervical lymph nodes draining intracerebral tumors in the mouse acquire a phenotype characterized by high expression of the two integrins α4 and α1 as well as a modest increase in the expression of P-selectin ligand [12]. Functional data supports a role for P-selectin/E-selectin and their ligands (including CLA) as well as integrin α4β1 (VLA-4) in extravasation of lymphocytes in the CNS during experimental autoimmune encephalomyelitis (EAE), but the relevance of these findings for trafficking of T cells across non-inflamed vessels during immune surveillance of the healthy brain remains unclear [13-16]. T cells in the CSF of healthy individuals or patients with non-inflammatory neurological diseases (NIND) are characterized by high expression of integrin αLβ2 (LFA-1), α4β1, CCR5 and CXCR3 [17,18], a phenotype they share with activated T cells isolated from a wide range of other organs [19-22]. Therapeutic α4-integrin blockade suppressed access of several cell types (CD4>CD8 T cells; CD19+ B cells; CD138+ plasma cells) into CSF [23], identifying the first functional CSF entry determinant for human lymphocytes.

In this study we addressed the expression of trafficking determinants known to be involved in organ-specific trafficking to the skin (CLA and CCR4) and the small intestines (integrin α4β7 and CCR9) on CSF T cells from patients with NIND. We reasoned that any bias in CD4+ T cell expression of these known organ-specific trafficking determinants in the CSF might aid in identifying CNS-specific adhesion molecules or chemokine receptors. By contrast, equal presence of tissue-committed T cells in the blood and CSF would indicate that memory T cells enter CSF according to their activation or differentiation state but possibly not by specific homing molecules. We observed comparable frequencies of CD4+ memory T cells displaying CLA and CCR4 as well as integrin α4β7 and CCR9 in paired blood and CSF samples. The results demonstrated that, in contrast to most other tissues, skin- and gut-homing T cells are not excluded from the CSF, suggesting that the recruitment of CD4+ T cells to the CNS is more related to previous antigen exposure and activation than to the expression of an organ-specific adhesion molecule or chemokine receptor. Although negative with regard to roles for CCR9/α4β7 or CCR4/CLA, for CSF homing, the results carry implications for the use of novel immunotherapeutic strategies to treat neurological diseases. In particular, several new techniques for treating diverse neurological disorders require the generation of neuroantigen-specific T cells by peripheral administration of antigen either subcutaneously or to the gastrointestinal mucosa. Our current results indicate that skin- and gut-homing memory T cells readily access the CNS compartment, providing a rationale for the feasibility of such strategies.

## Results

### CD4+ memory T cells expressing CLA and integrin α4β7 are present in the CSF

**Figure 1:**
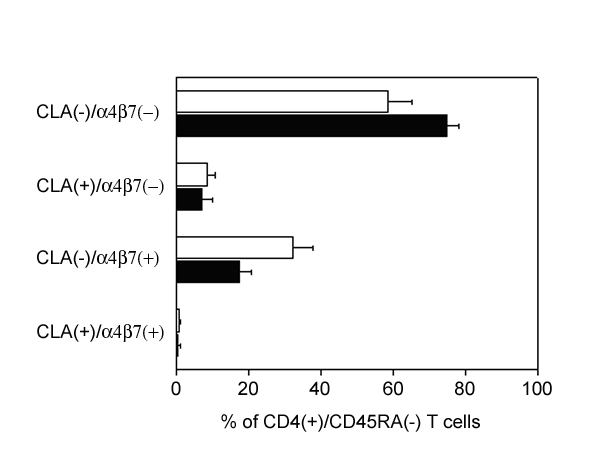
The majority of CD4+/CD45RA- memory T cells in the CSF (black bars) were CLA-/α4β7-, but discrete populations of CLA+ (associated with skin-homing) and integrin α4β7+ (associated with gut-homing) cells were observed. The expression pattern of CLA and integrin α4β7 was comparable on CD4+/CD45RA- memory T cells in peripheral blood (white bars) and CSF (black bars). Data are from four patients with non-inflammatory neurological diseases and figure shows mean+SEM.

Flow cytometry was used to determine the expression of CLA and integrin α4β7, two adhesion molecules involved in the selective recruitment of T cells to the skin and gut, respectively (reviewed by [1]), on T cells in paired blood and CSF samples from four NIND patients. Since many trafficking determinants are differentially expressed on naïve and memory T cells and CSF contains predominantly CD4+ T cells, of which almost all display a phenotype consistent with previously activated memory cells [18], we compared the expression of CLA and integrin α4β7 on CD4+/CD45RA- cells (excluding both CD45RA^hi ^and CD45RA^intermediate ^cells). The majority of CD4+/CD45RA- T cells in the CSF did not express either CLA or integrin α4β7 (74.9 ± 3.2%, mean ± SD). Discrete subpopulations of CD4+/CD45RA- T cells expressing either CLA (7.2 ± 3.0%) or integrin α4β7 (17.4 ± 3.5%) were, however, detected in the CSF. Interestingly, the pattern of CLA and integrin α4β7 expression on CD4+/CD45RA- memory T cells in the CSF was strikingly similar to peripheral blood (Figure 1), demonstrating that skin- and gut-homing T cells are not excluded from the CNS compartment.

### Expression of CCR4 on CSF T cells

**Figure 2:**
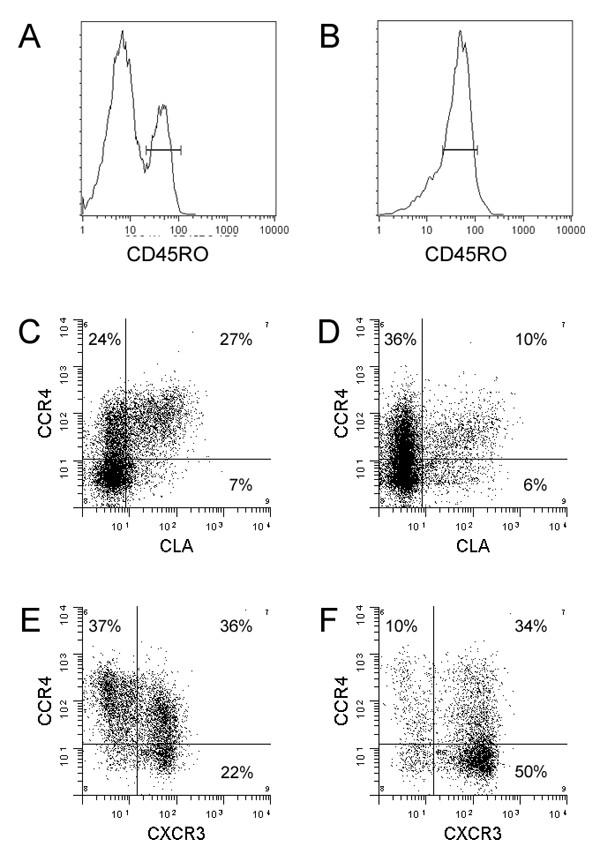
Multi-color flow cytometry was used to analyze the co-expression of CCR4 with CLA or CXCR3 on memory T cells in paired blood and CSF samples from an individual patient with a non-inflammatory neurological disease (A-D) or MS (E-F). The expression of trafficking determinants was analyzed on CD4+/CD45RO+ memory T cells to account for the different percentages of naïve and memory CD4+ cells in blood (A) and CSF (B). As expected, there was an association between the expression of CCR4 and CLA on CD4+/CD45RO+ T cells in peripheral blood (C). A majority of CLA+ memory T cells co-expressed CCR4 also in the CSF, but some CCR4 staining could be detected on CLA- memory T cells (D). While a large population of CCR4+/CXCR3- cells, which have been demonstrated to be enriched for Th2 cells [27], were present in peripheral blood (E), such cells were rare in the CSF (F).

Next, we assessed CSF expression of CCR4, a chemokine receptor highly expressed by skin-homing T cells [5]. Due to technical reasons, we used the presence of CD45RO to determine the population of memory CD4+ T cells for all CCR4 stainings (Figure 2). CCR4 was expressed by 41.7 ± 8.2% of CD4+/CD45RO+ memory T cells in the CSF from patients with NIND (Table 1). Consistent with a shared homing profile to the skin, a majority of CLA+ memory T cells in the CSF co-expressed CCR4, but some CCR4 staining could be detected also on CLA- memory T cells (Figure 2). Numbers of CCR4+/CD4+/CD45RO+ T cells in the CSF were slightly lower than in paired blood samples (59.8 ± 10.8%), but this difference was not statistically significant (n = 5; p = 0.09).

**Table 1:** Expression of trafficking determinants on CD4+ memory^1 ^T cells in blood and CSF

		CLA		Integrin α_4_β_7_		CCR4		CCR9	
		Blood	CSF	Blood	CSF	Blood	CSF	Blood	CSF
NIND	Mean	14.2	11.5	33.0	17.9	59.8	41.7	8.0	5.1
	SD	6.1	4.3	5.9	3.0	10.8	8.2	2.8	4.3
	n	10	10	4	4	5	5	8	8
									
MS	Mean	n.d.	n.d.	n.d.	n.d.	69.6	48.4	n.d.	n.d.
	SD					11.5	15.3		
	n					6	6		

^1^CD45RO+ or CD45RA- depending on staining protocol

NIND = non-inflammatory neurological diseases; MS = multiple sclerosis; CLA = cutaneous leukocyte antigen

Since CCR4 has been associated with a Th2 phenotype [24,25], CSF levels of CCR4 were analyzed in six patients with multiple sclerosis (MS), a disease associated with Th1 responses in the CNS [26]. MS patients had slightly lower numbers of CCR4+/CD4+/CD45RO+ T cells in the CSF compared to paired blood samples (Table 1; p < 0.01), but CCR4 was still expressed by approximately 50% of all CD4+/CD45RO+ T cells in the CSF. By carefully analyzing the profile of chemokine receptors on Th1 and Th2 cells, it has been demonstrated that Th2 cells predominantly are CCR4+/CXCR3-, while the population of CCR4+/CXCR3+ memory T cells not is enriched for Th2 cells [27]. We observed that a majority of CCR4+ memory T cells in the CSF co-expressed CXCR3 and that the numbers of CCR4+/CXCR3- "true" Th2 cells in the CSF were low (Figure 2).

The expression of CCR4 on CSF T cells was assessed using two different anti-CCR4 mAbs, clones 328B and 1G1. Preliminary experiments demonstrated that clone 328B resulted in a distinct staining of CSF T cells as described above, whereas clone 1G1 failed to detect any CCR4+ T cells in the CSF (data not shown). To confirm the expression of CCR4 on CSF T cells, RNA was isolated from CSF cells obtained from one patient with mononuclear pleiocytosis during the follow-up after postinfectious myelitis. Using real-time rt-PCR, we were able to verify that CSF mononuclear cells expressed CCR4 mRNA at slightly higher levels compared to PBMCs from the same individual (data not shown).

Preliminary stainings showed that CCR10, another chemokine receptor expressed by skin-homing T cells [28], was only expressed by a negligible percentage of CD4+ memory T cells both in blood and CSF and was thus not investigated closer (data not shown).

### CCR9 expression on CSF T cells

**Figure 3:**
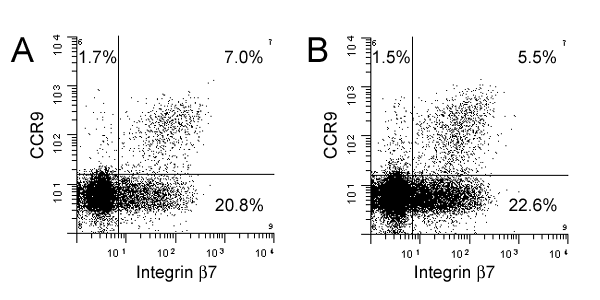
Flow cytometry was used to determine the expression of CCR9 and integrin β7, associated with homing to the gut, on CD4+/CD45RA- memory T cells in paired blood and CSF samples from patients with non-inflammatory neurological diseases. Memory T cells expressing CCR9 and integrin β7 were detected at comparable numbers in blood (A) and CSF (B), indicating that gut-homing memory T cells readily access the CNS.

Finally, the expression of CCR9, associated with homing of T cells to the intestines [6,7], was examined in the CSF. CCR9 was expressed by 5.1 ± 4.3% of CD4+/CD45RA- memory T cells in the CSF from patients with NIND, a frequency that was almost identical to peripheral blood (8.0 ± 2.8%; p = 0.1; Table 1). As expected, CCR9 expression was predominantly detected on CD4+/CD45RA- T cells co-expressing integrin β7 both in blood and CSF (Figure 3). Since the β7 subunit can dimerize not only with α4 (forming the α4β7 heterodimer, which is associated with gut-homing) but also with integrin α_E _(CD103), we stained CSF cells using an antibody against α_E _to exclude that CCR9 was associated with integrin α_E_β7 in the CSF. Virtually no CD4+/CD45RA- T cells in the CSF were, however, positive for α_E _(data not shown), confirming that gut-homing CCR9/α4β7 double positive memory T cells are present in the CSF.

## Discussion

This study demonstrated that CD4+ memory T cells in the CSF exhibit a distinctly different expression pattern of adhesion molecules and chemokine receptors compared to most tissue-infiltrating T cell populations. It is well established that T cells isolated from the small intestines and the skin are nearly homogenously positive for mutually exclusive pairs of trafficking determinants; almost all T cells from the skin express CLA and CCR4, but lack integrin α4β7 or CCR9, while T cells from the small intestines are positive for integrin α4β7 and CCR9, but not CLA or CCR4 [5,6,8,10]. This highly polarized pattern is believed to result from selective recruitment of T cells expressing the appropriate trafficking determinants from a pool of tissue-committed and non-committed T cells in the systemic circulation. It is plausible that additional tissue-committed T cell populations exist, since T cells from most other tissue compartments, including bronchoalveolar lavage (BAL) fluid and liver, do not express CLA, integrin α4β7, CCR4 or CCR9, suggesting that hitherto unknown combinations of adhesion molecules and chemokine receptors mediate organ-specific homing to these organs [9,10,29]. CSF from NIND patients contained, on the contrary, comparable numbers of CD4+ memory T cells expressing trafficking determinants specific for the skin (CLA and CCR4) and the small intestines (integrin α4β7 and CCR9) as paired samples from peripheral blood. Even though percentages of CD4+ memory T cells expressing CCR4 and integrin α4β7 were slightly lower in CSF compared to blood, the results demonstrate that skin- and gut-homing T cells access the CSF. These findings argue that T cell homing to the CSF is characterized by recruitment of a wide spectrum of tissue-specific T cell subpopulations. Aside from the established role of α4-integrin [23], the trafficking determinants involved in CSF trafficking remain to be defined.

These results address a key question regarding the feasibility of several experimental therapies for neurological conditions utilizing antigenic stimulation in the periphery (such as the skin or the gut) to produce a population of T cells, which are intended to act in the CNS. Such therapies include peripheral immunization against A-beta for Alzheimer's disease [30,31], sensitization with self antigens to generate myelin-reactive T cells for the amelioration of spinal contusion and neurodegeneration [32,33], as well as injection of altered peptide ligands [34,35] and oral administration of myelin to induce tolerance for the treatment of MS [34]. In recent years, such innovative approaches have been assessed in experimental animal models and in clinical trials, with provocative results [35].

To our knowledge, the only other tissue compartment besides CSF, which contains tissue-committed T cells specific for a different compartment is synovial fluid. Analogous to the present observations, synovial fluid from patients with arthritis contained comparable numbers of CD4+ memory T cells expressing CLA, integrin α4β7 and CCR4 as peripheral blood [10]. Both CSF and synovial fluid are sterile tissue fluids and the pathways for immune surveillance of such fluids may be different from the parenchyma itself. It has generally been assumed that cells in the CSF are derived from the brain parenchyma and reflect processes ongoing in the tissue compartment. Recent intravital microscopy studies have, however, demonstrated that leukocyte migration through the blood-brain barrier surrounding deep parenchymal vessels of the brain is an uncommon event in the non-inflamed murine brain [16], while fluorescently labeled splenocytes readily were detected in the meninges and choroid plexus within two hours after adoptive transfer [14]. Vasculature in the choroid plexus and meninges, but not in the non-inflamed human brain, express adhesion molecules supporting interactions with circulating leukocytes, suggesting that activated memory T cells may enter the CSF directly from the systemic circulation as part of immune surveillance of the CNS [36]. It is conceivable that in addition to the well characterized pathways of organ-specific homing to tissues in direct contact with the outside environment such as skin and gut, there may be distinct mechanisms controlling homing of antigen-experienced lymphocytes to sterile tissue fluids. The majority of CNS infections are derived from pathogens, which initially entered the body through mucosal sites or the skin, rendering it likely that memory T cells primed in these locations may mount the appropriate intrathecal immune response. In contrast, pathogens causing infections in the skin or the intestines are predominantly locally derived and a compartmentalized immune response may under these circumstances be phylogenetically beneficial.

## Conclusion

This study demonstrated that CLA+/CCR4+ and α4β7+/CCR9+ memory T cells are present in the CSF during immune surveillance of the CNS. Homing of T cells to the CSF during immune surveillance may be more tuned to distinguish between naïve and antigen-experienced T cells than among various tissue-specialized memory T cell populations. The ready access of skin- and gut-homing CD4+ T cells to the CNS compartment *via *CSF provide a rationale for the use of immunotherapeutic strategies, such as oral tolerance or therapeutic immunization, where immunogens are administered using an oral or subcutaneous route.

## Methods

### CSF samples

Blood and CSF were obtained from a total of 29 patients (20 women). The majority of the patients were referred for diagnostic lumbar puncture. In addition, three patients with known MS being evaluated for intrathecal baclofen treatment for intractable spasticity were included. The collection of blood and CSF samples was approved by the Institutional Review Board of the Cleveland Clinic Foundation (CCF) and written consent was obtained from all subjects. Because of limited cell numbers, the analysis of cell phenotypes in each sample was restricted to one or two trafficking determinants, along with cell lineage markers. The age of the patients ranged between 18–80 years (mean 43 years). When reviewing the charts, it turned out that 22 patients had non-inflammatory neurological diseases {NIND; headaches (6 patients), paresthesias (5), CSF circulation disturbances (3), chronic pain (3), vertigo (2), polyneuropathy (2), and mitochondrial cytopathy (1)}, six patients had clinically definite MS in remission [37], and one patient presented with pleiocytosis during follow-up after postinfectious myelitis. Three of the MS patients were treated with immunomodulatory drugs at the time of sampling (glatiramer-acetate or interferon-β1b). The CSF leukocyte count of the NIND patients ranged between 0–2 cells/μl (mean 0.8 cell/μl), while the CSF leukocyte count of the MS patients ranged between 2–41 cells/μl (mean 9.3 cells/μl). RNA was isolated from peripheral blood mononuclear cells (PBMCs) and CSF cells from the patient with postinfectious myelitis (CSF leukocyte count 13 cells/μl).

### mAbs

CD4 PerCP (clone SK3) and CD45RO APC (UCHL1) from BD Biosciences, San Jose, CA; β7 PE (FIB-504), CLA FITC (HECA-452), CXCR3 PE (1C6) and CD4 FITC/PE/APC (RPA-T4) from BD PharMingen, San Diego, CA; CD4 PE-Cy7 (SFCI12T4D11), CD45RA PE-TexasRed (2H4LDH11LDB9) and α_E _FITC (2G5) from Beckman Coulter, Fullerton, CA; CCR4 (328B) from ICOS, Bothell, WA; CCR9 (96-1) and α4β7 (ACT-1) from Millennium Pharmaceuticals Inc, Cambridge, MA; anti-mouse IgG1 PE/FITC from Southern Biotechnology Associates, Birmingham, AL; anti-mouse IgG Biotin and Streptavidin Cy5 from Jackson ImmunoResearch Laboratories Inc, West Grove, PA.

### Flow cytometry

Immunostainings for flow cytometry were performed as previously described [18]. Paired blood (5 ml) and CSF (10 ml) samples were collected and stained within 20 min of sampling. Staining was performed at room temperature for 15 min (CCR9, α_E_, β7) or on ice for 45 min (CCR4, CLA, α4β7). Titrations were performed for each mAb in blood samples to define the concentration that resulted in saturating conditions and an optimal signal to noise ratio. Identical concentrations of mAbs were used for blood and CSF samples, as numbers of CSF cells per staining never exceeded the number of cells in peripheral blood. Erythrocytes were lysed after staining whole blood using FACS lysing solution (BD Biosciences).

Cells were acquired on an LSR (BD Immunocytometry Systems, San Jose, CA) or a MoFlo flow cytometer (Cytomation Inc., Fort Collins, CO) and analyzed using WinList software (Verity Software House Inc., Topsham, ME). Cells were gated according to forward- and side light-scattering properties, and were positively selected for the presence of CD4 in combination with high expression of CD45RO (for all stainings containing CCR4) or the absence of CD45RA (for all other stainings) to identify CD4+ memory T cells. Isotype matched control mAbs were used to define background fluorescence.

### RNA isolation and real-time RT-PCR

RNA was generated from PBMCs isolated through density centrifugation on Ficoll (Lymphocyte separation medium; Meidatech Inc., Herndon, VA) and CSF mononuclear cells using Trizol (Invitrogen, Carlsbad, CA). Approximately 1 μg of DNase (Invitrogen) treated total RNA was reverse transcribed using Superscript II (Invitrogen) according to the manufacturer's instructions. PCR reactions were performed in 20-μl capillaries containing 2 mM Mg^2+^, 0.25 μl each of forward (5'-AAATGAACCCCACGGATATAGCAG-3') and reverse (5'-GAAAACACGAAGAGCAGATCCGAGA-3') primer [38], 1×DNA Master SYBR Green (LightCycler-DNA Master SYBR Green I kit; Roche, Indianapolis, IN) and 2 μl of cDNA using a LightCycler (Roche). Reaction conditions for PCR were as follow: denaturation at 95°C for 1 min, followed by 40 cycles of amplification by denaturation at 95°C for 15 s, annealing at 60°C for 5 s, and extension at 72°C for 30 s. The accumulation of products was monitored by SYBR Green fluorescence at the completion of each cycle. Construction of standard curves and analysis was performed with the LightCycler 3 software (Roche) as previously described [39].

### Statistical methods

Paired t-test was used for comparing expression of trafficking determinants on blood and CSF T cells. Reported p-values are two-tailed and considered statistically significant at p < 0.05.

## Competing interests

The author(s) declare that they have no competing interests.

## Authors' contributions

PK participated in the conception of the study, designed the experiments, participated in the performance of the experiments, analyzed the data, and drafted the manuscript. BT enrolled the patients in the study and carried out the flow cytometry assays. TW designed the molecular biological studies. JJC designed the 6-color immunofluorescent flow stainings and participated in the analysis and interpretation of the data. RMR participated in the conception and coordination of the study, contributed to the interpretation of the data and helped to draft the manuscript. All authors read and approved the final manuscript.
